# Tenosynovial giant cell tumours of the foot and ankle: a retrospective single centre experience with surgical treatment of 34 cases

**DOI:** 10.1186/s12885-025-13921-7

**Published:** 2025-03-23

**Authors:** Christian Scheele, Norbert Harrasser, Simone Beischl, Dietmar Dammerer, Ulrich Lenze, Carolin Knebel, Rüdiger von Eisenhart-Rothe, Florian Lenze

**Affiliations:** 1 Department of Orthopaedics and Sports Orthopaedics, Technical University of Munich, Klinikum Rechts der Isar, Ismaninger Str. 22, 81675 Munich, Germany; 2 Department of Orthopaedics and Traumatology, Krems University Hospital, Krems, 3500 Austria

**Keywords:** TGCT, Tenosynovial giant cell tumour, PVNS, Pigmented villonodular synovitis, Soft-tissue tumour, Foot, Ankle

## Abstract

**Background:**

Tenosynovial giant cell tumour (TGCT) is one of the most prevalent soft tissue tumours of the foot and ankle. Although typically benign, it can exhibit locally aggressive behaviour. This study aims to evaluate the distribution, surgical management, and recurrence rates of localized (L-TGCT) and diffuse (D-TGCT) forms of TGCT.

**Methods:**

A retrospective study of 34 TGCT cases in the foot and ankle treated surgically between 2010 and 2023 was conducted. Inclusion criteria required a histologically confirmed diagnosis and a minimum potential follow-up period of 18 months. Patient demographics, radiological findings, surgical approach and recurrence rates were evaluated.

**Results:**

Of 34 cases, 61.8% were L-TGCT and 38.2% were D-TGCT. L-TGCT had a significantly shorter duration of symptoms (median: 6 months) than D-TGCT (36 months, *p* = 0.01) and affected significantly more females (76.2%; *p* = 0.013). Nineteen cases were intraarticular, and 15 cases extraarticular manifestationsMacroscopically complete resection was achieved in 95.2% of L-TGCT cases and 69.2% of D-TGCT cases (*p* = 0.037). The recurrence rate with limited follow-up was 26.7% for L-TGCT and 50.0% for D-TGCT (*p* = 0.263). Time to recurrence was 7.0 months for L-TGCT and 12.0 months for D-TGCT (*p* = 0.287). In 40% of these cases, therapeutic intervention was performed.

**Conclusion:**

In the foot and ankle, L-TGCT is more common in females, presents earlier after symptom onset, and has a higher rate of complete resection, whereas D-TGCT has a longer symptom duration and higher recurrence rates. It’s important for orthopaedic surgeons to weigh surgical margins against functional results, as stable outcomes appear to be attainable even in cases of residual or recurrent tumours.

## Background

Tenosynovial giant cell tumour (TGCT) is one of the most frequently occurring soft tissue tumours of the foot and ankle and was previously known as pigmented villonodular synovitis (PVNS) [1–3]. Nevertheless, with an annual incidence of 1 per 1.8 million, it is a rare disease, affecting the synovium, tendon sheaths, and bursae of small and large joints, and generally occurring in the third to fifth decades of life [4–7]. It typically presents as a monoarticular condition, with the foot and ankle region being the second most commonly affected area, accounting for up to 23% of cases, after the knee joint, which accounts for 53% [8–10].

Two distinct types of TGCT exist, the localized (L-TGCT) and the diffuse TGCT (D-TGCT), which can occur intra- and extraarticular [6, 11–13]. These types exhibit different clinical presentations and prognosis [9, 11, 14–20]. The diagnosis of L-TGCT or D-TGCT is based on radiological and intraoperative findings.

Although it is generally a benign process, TGCT can exhibit proliferative, locally aggressive behaviour, which may result in progressive joint degeneration by infiltrating bone and cartilage [21–24]. The relative scarcity of TGCT cases in the foot and ankle often limited sample sizes in previous studies [25]. The objective of this study is to present the experience of an orthopaedic tumour centre in the treatment of TGCT of the foot and ankle to further contribute to the understanding and clinical management of the disease.

## Materials and methods

### Study design and patient selection

This retrospective study was approved by the local ethics committee and included all patients with a histologically confirmed diagnosis of TGCT of the foot and ankle who were presented to our multidisciplinary tumour board between January 2010 and August 2023 and underwent surgical treatment at our institution. Patients were included if they had a histologically confirmed TGCT of the foot and ankle, underwent surgical resection at our institution, and had a minimum follow-up period of 18 months (February 2025).

### Patient characteristics and tumour classification

Various patient characteristics were evaluated, including age, duration of complaints, symptoms, sex, side, BMI, and radiological tumour size. The type of TGCT (localised or diffuse), the presence of bone involvement and the anatomical location (intra- or extraarticular) were also analysed based on preoperative MRI imaging and surgical reports. For intraarticular manifestations, the joint from which the tumour originated was recorded. With regard to extraarticular TGCT, the anatomical classification proposed by Toepfer et al. [2] was used, which distinguishes between the forefoot (phalanges), midfoot (metatarsals and small tarsus), hindfoot (talus and calcaneus), and ankle (distal tibia and fibula).

### Diagnosis and surgical treatment

The diagnostic and therapeutic approach was assessed, including whether a primary biopsy was performed, the type of surgical procedure (open vs. arthroscopic), and the macroscopic resection status.

### Follow-up and outcome assessment

The number and timing of radiological follow-up examinations were documented, along with corresponding findings (residual, recurrence, recurrence-free) and, if applicable, size progression (progressive, stable). All medical reports, digitised treatment documents, and available radiological examinations were analysed.

### Statistical analysis

Data were collected and analysed using Microsoft Excel 2016 (Microsoft, Richmond, WA) and DATAtab (DATAtab 2023: Online Statistics Calculator. DATAtab e.U. Graz, Austria). The Kolmogorov-Smirnov test was employed for the analytical testing of the normal distribution of metric variables. For comparison of normally distributed metric variables, the t-test for independent samples was utilized. Levene’s test was conducted to assess equality of variance. In case of non-normally distributed metric variables, the Mann-Whitney U test was employed. The Chi2-test was conducted to examine associations between two categorical variables. To verify a balanced frequency distribution of a dichotomous variable, the binomial test was applied. The Kaplan-Meier curve was employed to analyse recurrence-free survival, with the log-rank test used to assess differences between groups. The significance level was set at *p* = 0.05.

## Results

Of the 34 cases of TGCT in the foot and ankle, 23 occurred in female patients (67.6%) and 11 in male patients (32.4%). The left side was affected in 67.6% of cases, while the right side was involved in 32.4%. An overview of patient characteristics is presented in Table 1. Age, BMI, and tumor size followed a normal distribution (*p* = 0.753, *p* = 0.323, and *p* = 0.246, respectively), whereas the duration of symptoms at initial presentation did not (*p* = 0.016).

**Table 1:** Baseline and preoperative characteristics of the 34 patients

	L-TGCT (*n* = 21)	D-TGCT (*n* = 13)	Total (*n* = 34)
**Demographic Data**			
Age (years, mean ± SD)	32.2 ± 17.5	37.4 ± 12.2	34.2 ± 15.7
Sex (male/female)	5/16 (23.8%/76.2%)	6/7 (46.2%/53.8%)	11/23 (32.4%/67.6%)
Side (left/right)	15/6 (71,4%/28,6%)	8/5 (61,5%/38,5%)	23/11 (67,6%/32,4%)
BMI (kg/m², mean ± SD)	26.1 ± 5.4	28.1 ± 7.5	26.8 ± 6.2
**Symptoms & Diagnosis**			
Symptom duration (months, median [IQR])	6.0 [1.0–12.0]	36.0 [29.3–87.0]	10.5 [1.8–36.0]
Pain only (%)	11.1%	7.7%	9.7%
Swelling only (%)	11.1%	15.4%	12.9%
Pain + Swelling (%)	77.8%	76.9%	77.4%
**Tumor Characteristics**			
Tumour size (cm, mean ± SD)	3.0 ± 1.4	3.8 ± 2.1	3.3 ± 1.7
Intraarticular cases (%)	10 (47.6%)	9 (69.2%)	19 (55.9%)
Extraarticular cases (%)	11 (52.4%)	4 (30.8%)	15 (44.1%)
Biopsy before resection (%)	25.0%	27.3%	25.8%

At initial presentation, 9.7% of patients reported pain only, 12.9% reported swelling only, and 77.4% experienced both pain and swelling. Of the 31 patients initially treated at our clinic (3 initially treated at other institutions), 23 underwent primary resection (74.2%). In the remaining 8 cases (25.8%), the histopathologic diagnosis was first confirmed by biopsy to exclude malignancy.

In total, 21 cases (61.8%) were diagnosed with L-TGCT, while 13 cases (38.2%) were diagnosed with D-TGCT.

The duration of symptoms was significantly shorter in L-TGCT, with a median of 6.0 months, compared to 36 months in D-TGCT (*p* = 0.01). Age, BMI, and tumour size did not differ significantly between the two forms of TGCT (*p* = 0.356, *p* = 0.371, and *p* = 0.254, respectively).

Among the 34 cases, 19 (55.9%) were intraarticular, with most (73.7%) originating from the tibiotalar joint. 21.0% originated from the talocalcaneal joint, and one case originated from the proximal interphalangeal joint of the third toe (5.3%). The remaining 15 (44.1%) were extraarticular, primarily affecting the midfoot (46.7%) and forefoot (33.3%). In addition, there were two cases (13.3%) in the hindfoot, and one case (6.7%) in the ankle. Intraarticular involvement was slightly more common in D-TGCT (69.2%) than in L-TGCT (47.6%).

Overall, 33 patients underwent open resection, while one patient underwent arthroscopic synovectomy. In one case, secondary bone grafting from the proximal tibia was performed 14 months after resection of a nodular TGCT in the anterior capsule of the tibiotalar joint to address osseus erosion at the neck of the talus.

The percentage of complete macroscopic tumour resection was 95.2% for L-TGCT, which was significantly higher than 69.2% for D-TGCT (*p* = 0.037; Table 2.). Bone involvement was observed in a total of 9 of 34 cases (26.5%), with 19.0% in L-TGCT and 38.5% in D-TGCT (*p* = 0.212).

**Table 2:** Macroscopic intraoperative tumour persistence and bone involvement in relation to the type of TGCT

		L-TGCT		D-TGCT		Total
		n	%	n	%	n
Complete tumour removal	Yes	20	95.2%*	9	69.2%*	29
	Unclear	1	4.8%	4	30.8%	5
Bone involvement	Yes	4	19.0%	5	38.5%	9
	No	17	81.0%	8	61.5%	25
	Total	21	100%	13	100%	34

**Fig. 1:**
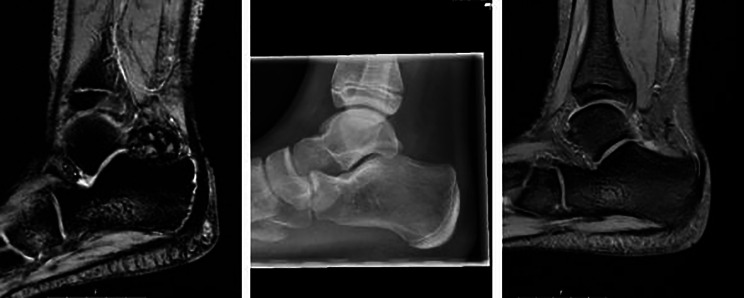
Case of L-TGCT with complete resection, showing preoperative MRI (sag hemo), preoperative X-ray, intraoperative specimen, and 3-year postoperative MRI confirming no recurrence

During follow-up 2 residuals and 8 local recurrences were observed after a mean time of 9.5 months (SD 6.0 months). In D-TGCT, 4 recurrences were observed in 8 initially tumour-free patients (50.0%) and in L-TGCT, 4 recurrences were observed in 15 initially tumour-free patients (26.7% Fig. 1) (*p* = 0.263, Table 3)

**Table 3:** Rate of recurrence in relation to the type of TGCT

		D-TGCT		L-TGCT		Total
		n	%	n	%	n
Recurrence	Yes	4	50.0%	4	26.7%	8
	No	4	50.0%	11	73.3%	15
	Total	8	100%	15	100%	23

**Fig. 2:**
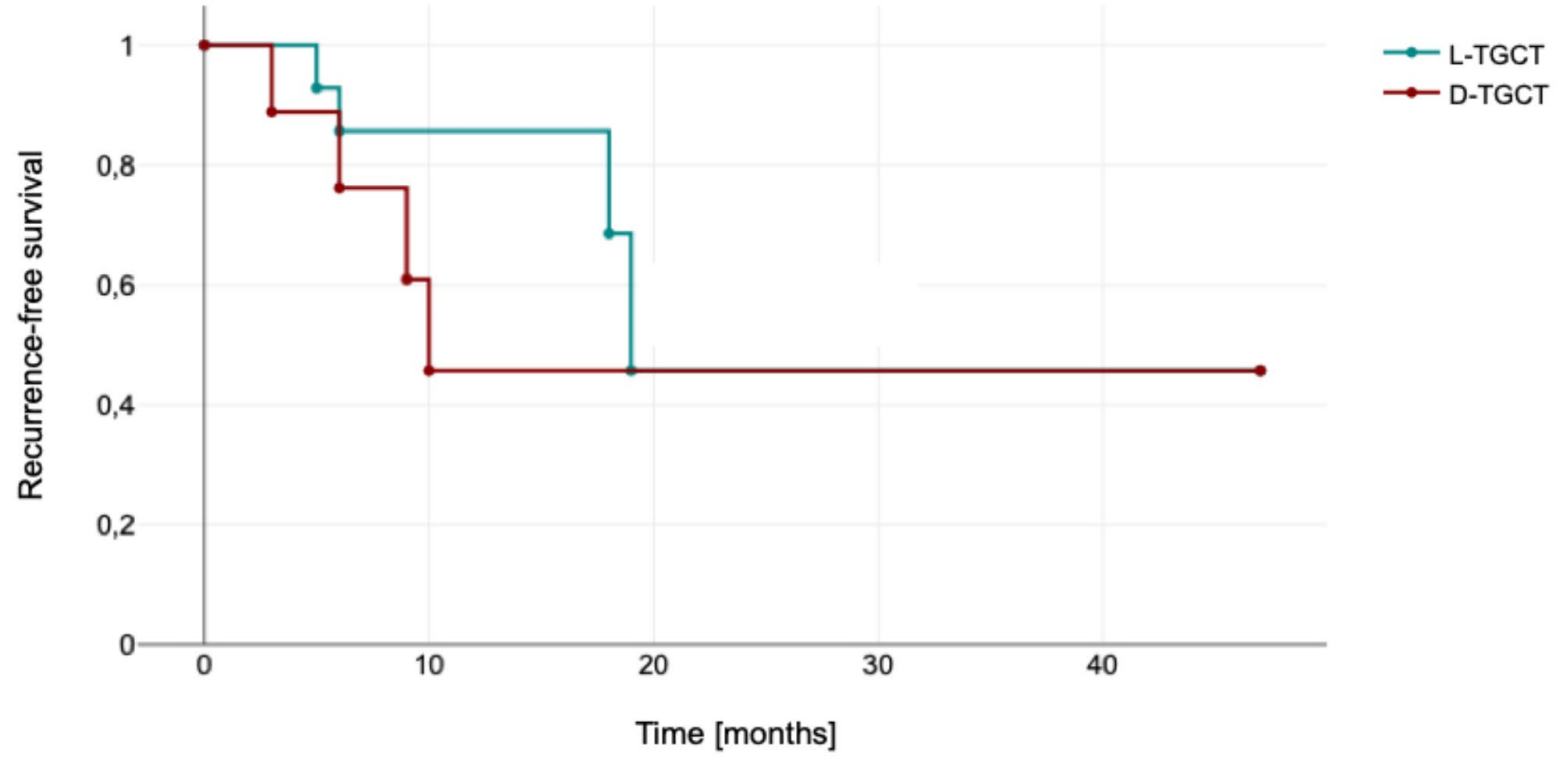
Kaplan-Meier survival curve for L-TGCT and D-TGCT

For D-TGCT, recurrences occurred at a mean of 7.0 months (SD 3.2 months; range 3–10 months) and for L-TGCT at a mean of 12.0 months (SD 7.5 months; range 5–19 months) (*p* = 0.287). Figure 2 illustrates this tendency for recurrence to occur earlier with D-TGCT.

**Fig. 3:**
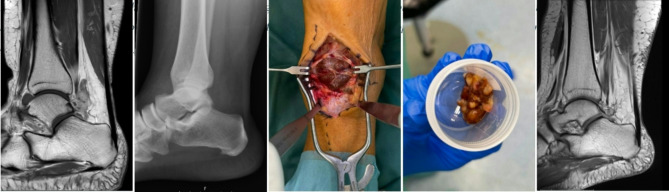
Case of D-TGCT with complete resection, showing tumor recurrence on 12-month follow-up MRI, necessitating revision surgery. From left to right: preoperative MRI (T1 sag KM), preoperative X-ray, intraoperative resection (ventral), resected specimen, and postoperative follow-up imaging revealing dorsal recurrence

Of the ten residuals and local recurrences, three cases underwent surgical revision after an average of 4.6 years (SD 4.1 years; range 2.0-9.3 years; Fig. 3) and one case underwent radiation after 23 months. In another case, the tumour board recommended radiation therapy following incomplete primary resection of a D-TGCT, but this was ultimately not performed. In the remaining 6 cases, clinical and radiological follow-up did not show any significant progress.

## Discussion

Diagnosing and treating TGCT of the foot and ankle remains challenging due to the relatively small number of patients and the heterogeneous presentation of the disease. In recent years, treatment options beyond surgery, including radiotherapy and systemic therapies have been explored, leading to an ongoing scientific debate [6, 20, 25–30]. However, open surgical resection remains the gold standard, while arthroscopic resection, radiotherapy or targeted systemic therapies may be beneficial in selected patients, for example in recurrent or unresectable cases [6, 20, 26, 30–33].

This study represents one of the largest single-centre series of TGCT of the foot and ankle. In accordance with previous studies, the patient population studied exhibited a prevalence of TGCT in the 3rd to 5th decade of life [18, 28, 34–37]. The mean age of patients with D-TGCT was slightly younger than that of patients with L-TGCT, at 32.2 years compared to 37.4 years. This was comparable to the results of a recent meta-analysis by Siegel et al. [25], which reported mean ages of 31.2 years and 36.6 years, respectively. The patient population studied demonstrated a female preponderance exceeding 60%, although a significant difference was observed solely within the L-TGCT subgroup (76.2% females vs. 23.8% males). This predisposition for female gender in L-TGCT of the foot and ankle is consistent with previous studies [36, 38–40], although it differs from single reports by Cevik et al. [41] and Cattelan et al. [42], in which 80% and 75% of patients with L-TGCT were male, respectively. Pain and local swelling were more frequently observed in our surgically treated patients compared to a study by Barnett et al. [20]. Considering the 2022 consensus paper recommending active surveillance for asymptomatic patients, this may well be considered an appropriate patient selection for surgery [27]. It is noteworthy that the time from symptom onset to medical presentation was significantly shorter for L-TGCT with a median of 6 months than for D-TGCT with a median of 36 months. In addition, nearly 10% of the patients studied underwent external surgery and were subsequently surprised by the histopathological result of a TGCT. In light of the potential diagnostic utility of MRI, this work should contribute to a heightened awareness of TGCT, thereby enabling a larger proportion of appropriate preoperative diagnoses in the future [43]. For diagnostic certainty, a biopsy was performed prior to resection in 8 of the 31 patients (25.8%). In our management, a biopsy is always performed when the radiological findings do not allow a diagnosis to be made with sufficient certainty and a malignant entity is to be ruled out.

Of the 34 lesions studied, 61.8% were L-TGCT and 38.2% were D-TGCT. This aligns with the most comprehensive retrospective series of TGCT of the foot and ankle, which reported a 69.1% prevalence of L-TGCT and a 30.9% incidence of D-TGCT [20]. In contrast, a meta-analysis of TGCT of the foot and ankle revealed that L-TGCT represents only 45.5% and D-TGCT 55.5% of the 382 patients included [25]. In our study, L-TGCT was observed intraarticular (*n* = 10) and extraarticular (*n* = 11) in approximately equal proportions, while D-TGCT was diagnosed intraarticular (*n* = 9) twice as often as extraarticular, which is in agreement with the results of Spierenburg et al. [30]. The 19 intraarticular lesions were mainly located in the tibiotalar joint (73.3%), while the extraarticular cases were more commonly found in the midfoot (46.7%) and forefoot (33.3%). The observed anatomical distribution pattern is consistent with previous studies [6, 20].

Macroscopically complete tumour resection was observed to be significantly less frequent in D-TGCT (69.2%) than in L-TGCT (95.2%), which is consistent with previous studies in other anatomic regions [11, 14–17]. Furthermore, bony erosions were observed with greater frequency in D-TGCT (38.5%) than in L-TGCT (19.0%), although the difference did not reach statistical significance. Palmerini et al. [9] demonstrated the clinical importance of macroscopically complete resection associated with superior local control. However, only 16% of the lesions examined in this study affected the foot and ankle. Tsukamoto et al. [44] were the first to demonstrate in a series of patients with hindfoot TGCT that macroscopically complete resection was associated with a reduced local recurrence rate. For this reason, given the complex anatomy of the foot and ankle and the proximity of neurovascular structures, our practice favours the open approach. However, studies have reported successful arthroscopic treatment of well-accessible lesions [28, 30, 45, 46].

Although clinical and radiological follow-up examinations were explicitly recommended, there were no follow-ups in more than one-quarter of cases. Furthermore, in almost two-thirds of cases, follow-up consisted of only one or two examinations, significantly limiting the power of the analysis of recurrence behaviour. The patient population, which is predominantly young and active, may have contributed to the poor adherence to follow-up recommendations. This is due to the benign nature of the tumour and the often slow progression of recurrence and residual disease. Additionally, patients may have expected to self-detect recurrence by symptoms such as swelling or pain without structured radiologic follow-up [23]. There is a lack of consensus in the literature regarding the optimal follow-up protocol. For instance, two studies recommended a follow-up period of 5 years with a reduction in the frequency of follow-up examinations over time [38, 44]. In contrast, other studies employed diagnostic imaging based on clinical symptoms [36, 47, 48] or relied solely on clinical symptoms [45]. In their meta-analysis, Siegel et al. [25] propose an initial structured clinical and radiological follow-up over 5 years for D-TGCT (baseline after 3 months, semi-annual follow-up for 2 years, and annual follow-up thereafter) and over 3 years for L-TGCT (baseline after 3–6 months, and annual follow-up thereafter). They recommend subsequent individualised follow-up depending on clinical symptoms.

The observed recurrence rate was 34.8% overall, with 50.0% for D-TGCT compared to 26.7% for L-TGCT, although this difference did not reach statistical significance. Tsukamoto et al. [44] reported a comparable overall recurrence rate of 30.3%. However, our findings should be interpreted cautiously due to the limited sample size and incomplete follow-up data. In the foot and ankle, a recent meta-analysis reported a recurrence rate of 28% for D-TGCT after open resection, and 7% for L-TGCT [25]. The higher recurrence rates observed in our study compared to some published literature may reflect selection bias, as symptomatic patients were more likely to attend follow-up appointments. A considerable proportion of patients, potentially those without symptoms, did not comply with recommended follow-up protocols, which represents a significant limitation of our study and may have led to an overestimation of recurrence rates. Furthermore, other studies have reported recurrence rates of 43–64% for D-TGCT [30, 37, 42, 44, 48] and 20–33% for L-TGCT of the foot and ankle [39, 42, 48].

In our cohort, the observed time to recurrence for D-TGCT averaged 7.0 months compared to 12.0 months for L-TGCT, though this difference was not statistically significant. These intervals are considerably shorter than those reported by Siegel et al. [25], who documented recurrences occurring after an average of 28.3 months for D-TGCT and 44.9 months for L-TGCT [25]. Our shorter observed time to recurrence may reflect our limited follow-up duration rather than true biological differences. It is important to note, however, that in both D-TGCT and L-TGCT, more than half of the recurrences are considered to occur within the first 24 months.

Ten cases of residual disease (*n* = 2) or local recurrence (*n* = 8) were observed. Therapeutic intervention was performed in only four cases. Furthermore, the mean time between primary resection and recurrence diagnosis was 1.0 years, significantly shorter than the time between primary resection and revision surgery, which was 4.6 years. These two observations demonstrate that TGCT often exhibits no or slow morphological and clinical progression, and that nonoperative management with symptomatic surveillance is a viable option. Barnett et al. [20] reported that more than three-quarters of 13 patients treated nonoperatively exhibited erosions but did not develop clinical symptoms. This is consistent with the observation that only 37–55% of cases after incomplete synovectomies develop recurrence of symptoms, and several studies have reported a regular lack of clinical and radiological progression [9, 14, 17, 20, 46]. Consequently, it is possible to achieve disease stabilisation, particularly when surgical resection is associated with significant loss of function, as TGCT is very rarely associated with non-local risk [9, 23, 30, 54].

Moreover, as TGCT is caused by an upregulation of the CSF1 gene and the recruitment of CSF1R-dependent inflammatory cells, tyrosine kinase inhibitors targeting the CSF1 receptor have been used to treat TGCT when surgery is not an option [49]. In 2019, the FDA approved pexidartinib for the systemic treatment of those adults with severe morbidity and functional limitations [50, 51]. In a real-world study, Lin et al. [52] reported clinically significant improvements in overall symptoms and physical function, as well as reduced use of medications and physical therapy during treatment. However, since its approval, only 0.4% of patients with TGCT in the US have been treated with pexidartinib [53] and other systemic therapies have been studied but are not specifically approved for the treatment of TGCT, such as anti-cytokine biologics and RANKL inhibitors [54, 55].

It is important to note that this study has several limitations. First, clinical data were collected from operative reports and correspondence, meaning that the quality of documentation could potentially affect the reliability of the results. Second, measuring the size of TGCT is challenging due to the complex morphology and heterogeneity of preoperative MRI images, which may have influenced the validity of this parameter. Third, assessing the completeness of resection in benign tumors is difficult as there is no histopathological confirmation of the resection status. As such, we relied on surgical reports to evaluate the quality of resection. Fourth, the overall number of patients and the number of patients with follow-up were relatively small, which limits the statistical power of the analysis. Some recurrences and recurrence-free courses were not identified due to the absence of routine MRI follow-up for all cases. Fifth, the retrospective nature of the study and the unstructured follow-up protocol introduce potential biases in our findings, and a comprehensive evaluation of complications and functional outcomes was not possible. Additionally, the single-center design limits the generalizability of our results to other clinical settings and patient populations. Nevertheless, this study provides valuable new insights into the understanding and management of TGCT of the foot and ankle.

## Conclusions

L-TGCTs are characterized by a shorter latency period from symptom onset to clinical presentation and exhibit a higher prevalence in females. Additionally, these tumors have a greater likelihood of complete resection. In contrast, our findings suggest that D-TGCTs may be more frequently associated with bone involvement and a higher risk of recurrence compared to L-TGCTs, although these differences did not reach statistical significance, likely due to the limited sample size. Intraarticular TGCTs predominantly affect the hindfoot, while extraarticular TGCTs are more commonly observed in the forefoot and midfoot. MRI generally provides an accurate diagnostic tool; however, in cases with ambiguous findings, biopsy remains essential for histological confirmation. Our results indicate that orthopaedic surgeons should carefully consider the balance between the extent of surgical resection and the preservation of functional outcomes. Despite some cases of residual or recurrent disease, stable clinical results may still be achievable, based on our limited follow-up.

## Data Availability

Availability of data and material: The datasets used and/or analysed during the current study are available from the corresponding author on reasonable request.
