# Correction: Tenosynovial giant cell tumours of the foot and ankle: a retrospective single centre experience with surgical treatment of 34 cases

**DOI:** 10.1186/s12885-025-14038-7

**Published:** 2025-04-07

**Authors:** Christian Scheele, Norbert Harrasser, Simone Beischl, Dietmar Dammerer, Ulrich Lenze, Carolin Knebel, Rüdiger von Eisenhart-Rothe, Florian Lenze

**Affiliations:** 1 Department of Orthopaedics and Sports Orthopaedics, Technical University of Munich, Klinikum Rechts der Isar, Ismaninger Str. 22, 81675 Munich, Germany; 2 Department of Orthopaedics and Traumatology, Krems University Hospital, Krems, 3500 Austria

**Correction**: ***BMC Cancer 25***,*** 530 (2025)***.

10.1186/s12885-025-13921-7.

Following publication of the original article [1], the authors identified that the author, Christian Scheele, was mistakenly displayed twice in the author group. The author group above has been updated and the original article [1] has been corrected.
